# Biodegradable and Compostable Plastics: A Critical Perspective on the Dawn of their Global Adoption

**DOI:** 10.1002/open.201900272

**Published:** 2019-12-17

**Authors:** Rosaria Ciriminna, Mario Pagliaro

**Affiliations:** ^1^ Istituto per lo Studio dei Materiali Nanostrutturati, CNR via U. La Malfa 153 90146 Palermo Italy; ^2^ Istituto per lo Studio dei Materiali Nanostrutturati, CNR via U. La Malfa 153 90146 Palermo Italy

**Keywords:** biodegradable, plastics, compostable, bioeconomy, megatrends

## Abstract

The global adoption of biodegradable and compostable plastics obtained from biomass‐derived monomers, we argue in this account, is now close to the inflection point. The first industrially significant impact will affect the packaging segment of the global chemical industry. In this process, China and India will play a pivotal role. Selected guidelines aiming to foster development of bioplastics industry in both developed and developing nations are provided.

## Graphical abstract

**Plastic** – **Quo vadis**? Estimates for biodegradable and compostable plastics market growth until 2025 suggest a modest yearly growth of less than 1 %. Yet, the bioplastics market will experience a production and uptake growth curve similar to that of photovoltaic solar cells since 2007, with China and India playing a pivotal role.

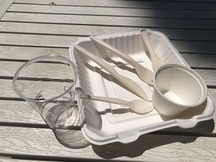

## 1 Introduction

The use of oil‐derived plastics – in particular that of the five main commodity thermoplastics (polypropylene, polyethylene, polyvinyl chloride, polystyrene and polyethylene terephthalate) – provides numerous and significant societal benefits.^1^ Used to package foodstuffs, water and beverages, for example, plastics contributes to the health and safety of people across the world.

Besides food, most personal care, cosmetic, and domestic products are packaged in plastic containers. Unfortunately, the chemical stability of these polymers, which is one of the main reason of their successful applications, gives rise also to serious environmental and health problems due to the huge amount of plastic waste released yearly in the environment. Thorough estimates in 2015 indicate that out of the overall amount of plastic waste produced between 1950 and 2015, only 9 percent was recycled.^2^ In the same year, around 55 percent of global plastic waste was discarded in the environment or landfilled, 25 percent was incinerated, and 20 percent was recycled.^2^

As a result, a minimum of 5.25 trillion plastics particles weighing close to 269,000 tonnes was conservatively estimated in 2013 float on the surface of world's oceans using an oceanographic model of floating debris dispersal.^3^

Similary, plastics pollutes the soil^4^ in the form of both macroplastics (>5 mm) and of microplastics, namely plastic particles <5 mm in size formed via macroplastics degradation, microbeads and microplastic fibres.^5^

Microplastics in soil and waters enters the food chain with detrimental health effects.^6^ Since 1991, and then even more since 2014, many nations have adopted policies to reduce single‐use plastic bags and to reduce plastic microbeads.^7^

In principle, biodegradable and compostable bioplastics would provide the aforementioned societal benefits while affording, respectively, lack of harmful residues or valued compost fertilizer.

Polylactic acid (PLA), starch, cellulose pulp, polyhydroxyalkanoates (PHAs) such as polyhydroxybutyrate, and polyhydroxyoctanoate are the main biopolymers used to produce today's single‐use bioplastics items such as bags, dishes, straws, coffee stirrers, glasses, horticulture pot, mulching film, bin‐liners, dust sheets, bottles and packaging items.

Reliable recent estimates suggest that plastic packaging accounts for as much as 40 per cent of the overall plastic consumption every year, largely dominated by petrochemical polymers.^8^

The first bioplastics after Rilsan (polyamide 11 or nylon 11) commercialized in the early 1950s, reached the marketplace in the early 1990s. Only recently, however, has their global adoption started to become significant, starting from single‐use bags, dishes and packaging applications. In 2018, bioplastics comprised close to one percent (2.112 million tonnes) of the about 335 million tonnes of global plastics production.^9^

Excellent books detail the chemistry and engineering aspects of bioplastics and biocomposites.^10^ In this study we argue that the global adoption of biodegradable and compostable plastics obtained from biomass‐derived monomers is close to the inflection point, with a forthcoming industrially significant impact due to shortly affect the packaging segment of the global chemical industry.

In this process, we further suggest in this account, China and India will play a pivotal role. We conclude providing selected guidelines aiming to foster development of bioplastics industry in both developed and developing nations.

## 2 From Plastic Waste to Fertilizers

In Europe, today's single‐use plastic packaging and lignocellulosic materials are biodegradable and compostable when meeting the requirements of the European standard EN 13432 “Requirements for packaging recoverable through composting and biodegradation – Test scheme and evaluation criteria for the final acceptance of packaging”.

Similar requirements for non‐packaging plastic items are specified by the European standard EN 14995.

**Figure 1:**
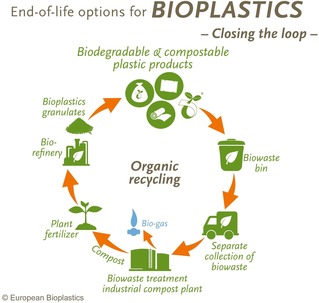
Recycling biodegradable and compostable plastic products afford compost (a valued fertilizer) and biogas. Compost is used to ferilize plant growth after which green chemistry processes at the biorefinery convert plant‐derived sugars or terpenes into new bioplastics. [Image courtesy of Europe Bioplastics].

In other words, the latter standards define the characteristics that a material must possess in order to be considered “compostable”, namely that it can be recycled through organic recovery (composting and anaerobic digestion, Figure 1).

EN 13432 requires that the following four characteristics are tested in a laboratory:

1. Disintegration, namely fragmentation and loss of visibility in the final compost ‐ this is measured in a pilot composting test in which specimens of the test material are composted with biowaste for 3 months. After this time, the mass of test material residues has to amount to less than 10 % of the original mass.
2. Biodegradability, namely the capability of the compostable material to be converted into CO_2_ under the action of microorganisms. The standard contains a mandatory threshold of at least 90 percent biodegradation that must be reached in less than 6 months (laboratory test method EN 14046).
3. Absence of negative effects on the composting process.
4. Amount of heavy metals has to be below given maximum values, and the final compost must not be affected negatively (no reduction of agronomic value and no ecotoxicological effects on plant growth). Plastics certified according to EN 13432 can be labeled by the “Seedling” logo (Figure 2
  **Figure 2:**
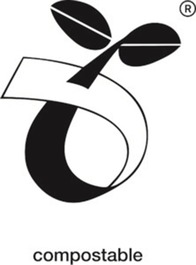
The Seedling label for compostability. [Image courtesy of Europe Bioplastics].
  ) helping consumers to correctly identify the compostable material assisting in the decision on purchasing and disposing a product.

Certified, compostable plastics including items in PLA are sent to industrial composting plants by disposal as organic waste. Indeed, contrary to what is frequently reported in the grey literature, PLA (a very versatile material and an excellent replacement for polystyrene and polypropylene in demanding applications) already produced at >100,000 t/a rate, is biodegradable and compostable.^11^

For example, degradation of PLA sheets under composting plant conditions studied in Thailand in 2008 shows that after 8 days under composting plant conditions at relatively high temperature and humidity (50–60 °C and RH 60 %) the sheets became brittle and started to break into small pieces.^12^

The typical temperature at a land composting plant is higher than the glass transition temperature of PLA, enabling penetration of water molecules through the disordered polymer chains, thereby enhancing the hydrolysis reaction.

Even at home, PLA which is not home compostable, when blended with polycaprolactone (PCL) becomes home‐compostable.^13^ In other words, bioplastics manufacturers can manufacture PLA‐based plastic blends which can be made home or industrial compostable, more or less rapidly biodegraded, depending on the use of the bioplastic objects commercialized.

As a result, compostable tableware and cutlery in PLA or in PHA used, for example, at large events (sports, concerts, ceremonies etc.) as well as in restaurants can then be disposed of together with the food waste in one single compostable “waste” stream. Waste, however, will turn into high quality compost/soil improver fertilizer and valued biogas according to the principles of the circular economy.

Composting takes place at the same composting plants, ubiquitous for example in Italy or in Germany, where food and other organic waste undergoes conversion into compost and biogas.

## 3 Economic and Industrial Aspects

To understand why bioplastics remained a niche of the overall plastics market even in low value‐added segment of packaging or bags, it is revealing to review the comments of an India's pioneer entrepreneur in bioplastics:

“Suddenly, in 2012, Mangaluru City Corporation imposed a ban on plastics, I thought that I must invest in this venture of providing cloth bags in Mangaluru. During the distribution of cloth bags, a fisherwoman came to me and asked me if I was the reason behind the ban on plastics. She raised a serious question when she asked me about how she could sell fish worth Rs 20 in a bag which costs Rs 25. No one was ready to buy these bags.”^14^

High production costs, and therefore high price, have prevented for more than two decades bioplastics from competing with petrochemical polymers. Today, however, it has been enough to deploy relatively modest investments in research and new production routes, to change the situation. The bags produced at the first manufacturing unit is located in Peenya, India, starting from corn starch, vegetable oil derivatives and vegetable waste now cost Rs 3, with the bags produced based on customer demand.^14^

In general, bio‐based polymers are usually produced in small plants in which either biopolymers such as starch are processed into thermoplastic polymers, or sugars are converted into polymers via fermentation routes. Manufacturing units are distributed across a country, almost in opposite fashion to large, centralized petrochemical plants where oil is first refined and then its fractions and molecules undergo through highly efficient heterogeneously catalyzed processes to produce chemicals and polymers.

Apparently, successful competition with petrochemical companies, accumulating large earnings from the sale of petrochemicals when the price of oil is low (i. e. <$40/b), and compensating losses from petrochemical manufacturing when the price of oil is high through the sale of fuels, would seem impossible.

This explains why in the last decade numerous companies targeting bioproducts such as bioplastics failed, were acquired by petroleum companies or changed productions targeting higher value products such as cosmetic ingredients.

However, the combination of low and rapidly declining EROI (energy returned on energy invested) for oil, demography and global economic growth demands some 32 additional million barrels per day by 2025.^15^

In other words, in less than a decade, for the global economy continuing to grow at natural pace, mankind should add more than one third of current 90 million barrels consumed daily.

Under these conditions, switching the production of chemicals and polymers from oil to biomass, and that of energy from fossil fuels to renewable energy sources, is inevitable prior to serious global energy and resource crisis.

According to a recent analysis commissioned by European Bioplastics (an association based in Germany representing about 70 members from the entire value chain of bioplastics), global production capacities of bioplastics are predicted to grow from around 2.11 million tonnes in 2018 to approximately 2.62 million tonnes by 2023.^16^ For comparison, the overall global plastic production of plastics in 2017 was 335 million tonnes, and since then it has continued to grow.

**Figure 3:**
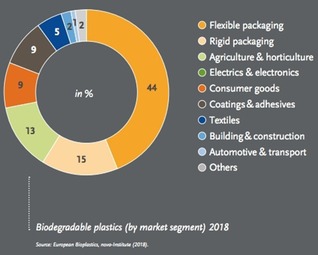
Biodegradable plastics (by market segment) 2018. [Image courtesy of European Bioplastics, 2019].

Biodegradable bioplastics are very well suited for packaging applications. Indeed, flexible and rigid packaging accounted for about 60 % of their market in 2018 (Figure 3), followed by agriculture and horticulture.

Estimates for biodegradable and compostable plastics market growth until 2025 suggest a modest yearly growth of less than 1 %. According to the aforementioned association's estimates, the global market would grow by a mere 24 % in five years (2018–2023).

Yet, we argue in the following, exactly as it happened with the unexpected massive production and uptake of solar photovoltaic modules for electricity production,^17^ China (and India) have a synergistic opportunity with switching from imported oil to locally sourced biomass to make bioplastics: clean their environment, improve public health and agriculture, and at the same time build a new profitable industry targeting the global market in which societies, and especially younger consumer generations, demand green and sustainable products posing no threats to health and to the environment.

## 4 The Pivotal Role of China and India

China and India currently account for about 37 % of the world's population, with China currently home to about 1.4 billion people and India to 1.3 billion.

Alone, the consumption of thin (4–8 μm) polyethylene sheets of Chinese farmers increased from 6,000 tonnes, covering 0.12 million ha, in 1982, to 1.2 million tonnes, covering almost 20 million ha, in 2011.^18^

Such thin film comprised of non‐biodegradable polyethylene is easily damaged, difficult to remove and cannot be reused for a second season. The outcome discovered in 2014 is that residual plastic in the top 0.3 m soil layer was estimated to vary from 72 to 260 kg/ha, depending on number of years use, percentage of ground covered and film thickness, significantly diminishing crop yields.^18^

Showing further evidence of the policy crucial role in promoting bioplastics adoption, after regulations to ban plastic bags in several Europe's countries including Italy, China in 2017 banned almost all foreign plastic waste imports, causing troubles to most urban waste recycling plants in Europe and in the USA.^19^

In mid 2019, China's president stressed once again the importance of pollution prevention and control for a truly sustainable economic growth.^20^ It may not be surprising to learn that in late 2018 a subsidiary of China's state‐owned and largest food company began production of biodegradable PLA resin. Similarly, a Chinese corn processing company along with a subsidiary of a France's sugar company is currently building thanks to an overall investment of about $100 million a PLA plant in Anhui Province with a capacity to produce 100,000 tonnes per year by 2020.^21^

At the same time, existing bioplastics companies in China are growing at fast rate. For example, the amount of PLA at one firm manufacturing the resin in the Zhejiang Province has gone from 5,000 tonnes in 2006 to 15,000 tonnes in 2018, with projected increase in production to 65,000 tonnes by 2020.^21^

Existing conventional plastics manufacturers in China are therefore quickly expanding their productions to include bioplastics manufacturing. For instance, a large conventional plastics manufacturer with installed production capacity of over 600,000 tonnes in 2017, plans to build an additional production capacity of 300,000 tonnes of “biological composite materials” in 2019, focusing at the beginning on bioplastics for packaging.^21^

In this context, India will not repeat what happened with its solar cell and photovoltaic module manufacturers which, likewise those based in Europe, make up today a negligible fraction of the current >100 GW global solar cell production, chiefly taking place in China.

Following the August 15, 2019 Independence Day address in which India's premier called for ban a single‐use plastic ban for six types of plastics, the expected ban was “put on hold, with officials stating the ban would be too disruptive to industry”.^22^

The aforementioned Bengaluru‐based company producing low cost biodegradable plastics bags already has it products available in 12 countries beyond India. Made from tapioca starch and vegetable oil, and affording a thermoplastic resin not requiring industrial composting conditions to biodegrade, the polymer rapidly decomposes even under ambient conditions.

Aptly called “unplastic” the resin, after passing boiling water, burning, hot iron, edible and strength tests conducted by public and private industrial product certification bodies, is currently undergoing the EN 13432 test for biogradable certification.^23^

The raw materials used to produce the resin (corn starch, potato, tapioca, corn, natural starch, banana, flower oil, vegetable oil derivatives and vegetable waste) are locally sourced at low cost, which along with the low cost production process, translates into unprecedented low prices for the resulting biodegradable bags.

The company is partnering with several other companies to start local production in the numerous States comprising the huge India nation, so as to scale up production, and cut transportation costs thereby exploiting the advantages of polymer distributed manufacturing in place of the centralized petrochemical production.

India's agriculture today is so developed that the country not only satisfies the food needs of its 1.3 million inhabitants but also exports large amounts of crops (for example, out of the 90.8 million tonnes of wheat produced in the country, in 2014–15, 29 million tonnes was exported).^24^ Agricultural, forestry and food processing industry by‐products such as for example molasses and sawdust are ideally suited to source both polymers and sugars to manufacture new generation bioplastics.

In this way, not only bioplastics manufacturing will not deprive the production of food, but it will actually provide extra revenues to India's farmers who, in their turn, will benefit from using mulch bioplastics in place of the polyethylene sheets or bisphenol‐based polycarbonate for their greenhouses.

Indeed, providing a comprehensive perspective on the need to transform the linear plastics economy into a circular plastics economy, Lin and co‐workers have lately shown how waste valorization through green chemistry technology can practically realize the concept of a plastics circular economy through for example a biorefinery to produce high value‐added fructose via food waste bioconversion,^25^ or via textile waste biorefining to produce valued glucose syrup and polyester.^26^

## 5 Conclusions

Driven by societal megatrends concerning the environment, health, and energy which permeate societies on a global scale, significant product and process innovation is taking place in the chemical industry.^27^ These megatrends include a profound reshape in customer demand in which lack of negative impact on the environment and human health of chemical products, starting from plastics, becomes central.

It is this change which is driving rapid advancements and innovations in bioplastics manufacturing, including the discovery of new bioplastics‐based materials with improved properties and new functionalities in sight of their wide uptake in sectors today dominated by petrochemical polymers such as textiles, construction and building, consumer goods, and automotive applications.

New and mass demand of biodegradable and compostable bioplastics, already unfolding in China, will shortly arise also in India. Together, the world's two most populated countries are eminently suited for large‐scale bioplastics manufacturing, exactly as it is happening for widespread distributed energy generation thanks to photovoltaic solar energy.^17^, ^28^

In this rapidly evolving context, in which the bioplastics market growth will not be limited to 1 or 2 per cent but it will experience a curve more similar to that of photovoltaic global uptake after 2007, the other countries owning large chemical companies will necessarily start to enter bioplastics manufacturing. It is instructive, in this respect, to notice that in Japan “in the last year or so… local manufacturers are turning to biodegradable plastics and specialty paper products as an alternative to single‐use plastic packaging”.^29^

Given the intrinsic nature of the bioeconomy relying on biological and renewable resources, all world's countries may benefit from establishing their own bioplastics industry. To foster progress towards this aim, two guidelines emerge from the present study.

First, existing and new bioplastics companies need to increasingly adopt highly efficient, continuous production technologies largely based on heterogeneous biocatalysis similar,^30^ even though on different scale and under much milder reactions conditions, to those based on heterogeneous chemocatalysis employed by the petrochemical industry.

Second, to increase knowledge creation and its transfer to the new industry, and address the shortage of skilled workforce and researchers, countries should proactively act by establishing new bioeconomy research and educational institutes able to give also more useful policy advice.^31^, ^32^

Critics of renewable energy first pointed to its “unbearable high economic cost” and then, when the cost of generating electricity using PV modules became by far the cheapest among all energy sources, they emphasized the “intermittent and intrinsically unreliable nature” of energy generate by sunlight or wind. Even in a remote Hawaii's island only this year one utility company will save purchase and import of more than 1,000 tonnes of diesel fuel, supplying its customers with electricity of unsurpassed quality (frequency of the alternating current, and stable voltage) generated with PV modules stored in a 100 MWh battery energy system.^33^

Chemical companies in conventional plastics may wish to learn from this single example how to avoid what happened to the manufacturers of turbines for thermoelectric power plants, whose global market, displaced by the world's adoption of PV and wind power, has gone from a total generation capacity of 71.6 GW in 2011 to about 30 GW in 2018,^34^ and is currently continuing to shrink.

## Conflict of interest

The authors declare no conflict of interest.
